# Hydroxycarbamide‐related cutaneous ulcer complicated by osteomyelitis

**DOI:** 10.1002/jha2.309

**Published:** 2021-10-01

**Authors:** James T. England, Vikas Gupta

**Affiliations:** ^1^ Division of Medical Oncology and Hematology Princess Margaret Cancer Centre Toronto Ontario Canada


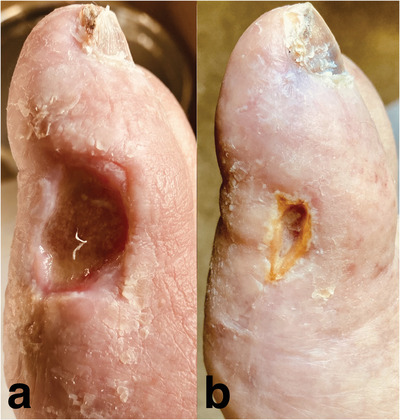


A 78‐year‐old male with a history of polycythemia vera (PV) presented with bilateral foot ulcerations (a) limiting his ability to exercise or wear shoes. His PV had been managed for the previous 5 years with aspirin 81 mg per os (PO) daily, intermittent phlebotomy, and hydroxycarbamide (hydroxyurea [HU]) 1500 mg PO daily. At the time of presentation, his hemoglobin was 138 g/L, hematocrit 0.41, white blood cell count 14.5 × 10^9^/L, and platelet count 695 × 10^12^/L. Cultures from the right toe ulcer grew *Pseudomonas aeruginosa* and *Streptococcus pyogenes*; scintigraphy bone scan demonstrated evidence of osteomyelitis of the right first phalanx. The HU was held and the patient was started on a 6‐week course of ciprofloxacin and doxycycline. Ruxolitinib 10 mg PO BID was initiated for management of PV. Follow‐up 3 months later demonstrated significant improvement of the cutaneous ulcerations (b).

Cutaneous toxicity can occur in 5%–12.5% of myeloproliferative neoplasm patients treated with HU, and can lead to significant complications such as the secondary osteomyelitis seen in this case. Etiology of ulcers is multifactorial with microvascular disturbance from reduced red blood cell deformability and reduced wound healing due to cytotoxic effects on keratinocytes and basal cells of the epidermis and endothelium. European Leukemia NET (ELN) criteria for HU intolerance includes development of lower extremity ulcers and should prompt the use of alternative cytoreductive therapy. Patient counselling and clinician recognition of HU cutaneous adverse events may allow for rapid therapy change and prevention of complications.

## DECLARATION OF CONSENT

Patient provided written consent for photographs to be used for publication and education purposes.

## Data Availability

Data sharing is not applicable to this article as no new data were created or analyzed in this study.

